# The Clinical Applications of Psilocybin Therapies and Post-COVID Syndrome: A Comprehensive Narrative Review

**DOI:** 10.7759/cureus.86659

**Published:** 2025-06-24

**Authors:** Alfred Mathew, Roshan Dongre, Seo Hee Kim, Jack Turner, Alen Mathew, Ellie Cherryholmes, Millad Mehrinfar, Sagar Kamprath

**Affiliations:** 1 Department of Psychiatry, University of Louisville School of Medicine, Louisville, USA; 2 Department of Engineering Medicine, Texas A&M College of Medicine, Houston, USA; 3 Department of Pediatrics, University of Texas at Austin Dell Medical School, Austin, USA; 4 Department of Internal Medicine, Oklahoma State University College of Health Sciences, Tulsa, USA; 5 Department of Neurology, Örebro University Hospital, Örebro, SWE; 6 Department of Pharmacology and Toxicology, University of Texas Medical Branch at Galveston, Galveston, USA; 7 Department of Psychiatry, University of Texas Medical Branch at Galveston, Galveston, USA; 8 Department of Family Medicine, Houston Methodist Hospital, Houston, USA

**Keywords:** alternative medicine, covid-19, mental health, post-covid syndrome, psilocybin

## Abstract

The coronavirus variant (causing the COVID-19 disease) that led to a pandemic sent global shockwaves, resulting in long-term effects on physical, mental, and social well-being and impacting both individuals and communities. With the pandemic’s notable impact on mental health, one such potential treatment discussed in recent literature is psilocybin. Psilocybin is a naturally occurring prodrug compound found in select mushrooms shown to reduce clinical symptoms of certain mental health disorders. In this study, we review the status and usage of psilocybin in clinical practice preceding and following the COVID-19 pandemic. The search criteria for the study included psilocybin or psychedelics or psychedelic-therapy psychiatry and long-haul COVID. The search spanned English articles from January 2020 to April 2024, utilizing the PsychInfo, Cumulative Index to Nursing and Allied Health Literature (CINAHL), Scopus, and PubMed databases. Two reviewers independently screened each record to decide if a study met the inclusion criteria and to account for bias. Each article researched different pathologies, including depression, anxiety, post-traumatic stress disorder, and COVID-19. The manuscripts collectively emphasize that there is evidence that psilocybin has a role in the treatment of said pathologies, with relatively safe outcomes if administered under proper medical supervision. Psilocybin use was followed up for a relatively long period after some trials, but further research is warranted to draw a more definitive conclusion regarding the therapeutic uses of psilocybin. Our review reflects that barriers to using psilocybin therapeutically for long-haul COVID-19 exist, which significantly impacts the scope of our research. While evidence suggests its efficacy in mental health conditions such as depression and mood disorders, more robust clinical trials are needed. Current literature supports the pharmacological basis that psilocybin may be effective in treating COVID-19 sequelae. Psilocybin’s role in inhibiting SARS-Cov-2 protease shows promise, but ultimately, in vitro validation will be necessary before wider approval of the drug. Lastly, large clinical trials comparing psilocybin to standard care and assessing symptom relief in long-term COVID patients may help validate the findings seen in much of the current literature.

## Introduction and background

The emergence of the coronavirus variant (that caused COVID-19) in early 2020 rapidly transformed the socio-political landscape worldwide. To safeguard public health, countries implemented dynamic mitigation strategies, placing significant strain on healthcare systems. Adequate staffing, bed capacity, and personal protective equipment for providers became critical concerns. In the United States, vulnerable populations, such as immunocompromised and obese individuals, faced heightened risks for COVID-19 severity. While some groups could engage in mitigation strategies, essential workers continued to work precariously, and nonessential workers were transitioned to virtual realms during strict lockdowns. The pandemic also affected public education, leading to remote classrooms and ongoing debates over how to handle the pandemic in local communities.

COVID-19 intersected with public health at social, medical, economic, and political levels. Before the pandemic, certain aspects of healthcare, such as mental health, were already in crisis. COVID-19 exacerbated this trend and created a significant impact on mental health in the general population. The long-term health implications of COVID-19 are still being identified and managed today. COVID-19 triggers a series of immunologic events that can lead to severe disease and mortality. Infection initiates a systemic inflammatory response syndrome that perpetuates a pro-inflammatory state. If compensatory anti-inflammatory response syndrome (CARS) systems cannot reduce this proinflammatory state, a “cytokine storm” may occur, followed by acute lung injury, acute respiratory distress syndrome, hypercoagulation, low blood pressure, organ failure, and mortality.

Conversely, an overresponsive CARS may lead to a highly immunosuppressed state, which is believed to contribute to the development of post-COVID syndrome (PCS) [1]. These are symptoms that persist over four weeks after the initial COVID-19 infection and may include fatigue, attention deficit, headache, hair loss, dyspnea, myalgia, arthralgia, and depression [2,3]. As of now, there is no known cure for PCS, and researchers are actively exploring alternative treatments.

One such treatment under investigation is psilocybin, a 5H2A serotoninergic agonist that has had a history of use in indigenous rituals and healing for over a millennium. Since its discovery by the Western world in the mid-1900s, its use has invited both controversy and clinical promise. In recent years, psilocybin’s potential therapeutic uses in conjunction with therapy for mood disorders such as depression, anxiety, and post-traumatic stress disorder (PTSD) have been explored in psychedelic-assisted therapy. Psilocybin’s serotonin activation and ability to induce profound “mystical experiences” have shown positive outcomes in patient cognition, behavior, and mood [3-7]. Combining these health benefits with a highly amicable safety profile and a low abuse profile makes psilocybin a strong candidate for future pharmacological intervention. Recent studies indicate that psilocybin administration promotes neuroplastic changes, which may have potential in treating the sequela of PCS [8,9]. This narrative review explores the intersection between psilocybin and selected health impacts associated with the COVID-19 pandemic while highlighting its potential in treating PCS.

Methods

Articles were included in the study if they were related to psilocybin and long-haul COVID. The search criteria for this literature review included the terms: “psilocybin,” “psychedelics,” “psychedelic-therapy,” “psychiatry,” “long-haul COVID,” “post-acute COVID-19,” “post-acute sequelae of SARS-CoV-2 infection”, or “post-COVID syndrome.” The search strategy was developed in consultation with a research librarian to ensure comprehensiveness. Articles were searched from January 2020 onwards and limited to English-language publications. The databases PsychInfo, Cumulative Index to Nursing and Allied Health Literature (CINAHL), Scopus, and PubMed were searched using Boolean operators. The final search was conducted on October 24, 2024. All retrieved records were imported into a reference management software, where duplicate records were identified and removed before screening. A total of 117 articles were identified. Screening was conducted in two stages: title and abstract screening, followed by full-text screening. Articles were evenly divided between reviewers for the initial screening stage.

Each reviewer independently screened titles and abstracts against the pre-specified inclusion criteria, which included a reference to psilocybin in the context of long-haul COVID. In cases where the relevance of an article was uncertain, the article was appraised by two additional reviewers. Discrepancies were resolved through discussion, and if consensus could not be reached, a fourth reviewer was consulted. After the initial screening, 89 articles were excluded for not meeting the inclusion criteria, and three duplicates were removed. The remaining 28 articles underwent full-text review. This process was conducted independently by multiple reviewers to ensure rigor. 

Following the full-text review, 21 articles met the inclusion criteria. During this process, articles were excluded for not aligning with the original search criteria, such as those focusing on psilocybin without a connection to COVID-19. Data from the included studies were extracted using a pre-piloted data extraction form. Two reviewers independently extracted data on study characteristics, including authors, publication year, study design, sample size, participant characteristics, intervention details, and outcomes related to psilocybin use in the context of long-haul COVID. Any disagreements in data extraction were resolved through discussion or consultation with a third reviewer. 

Two independent reviewers assessed the risk of bias across multiple domains, including bias in selection, performance, detection, attrition, and reporting. Discrepancies in bias assessment were resolved through discussion or consultation with a third reviewer. The findings from the risk of bias assessment were used to interpret the results and are discussed in the context of the overall conclusions. Given the heterogeneity of the included studies, a qualitative synthesis was performed. Data was synthesized narratively, focusing on reported outcomes related to psilocybin treatment for PCS. Due to the limited number of studies and variations in study design and outcome, a meta-analysis was not conducted. However, where appropriate, we examined the consistency of results across studies and explored potential sources of heterogeneity. Publication bias and selective reporting within studies were assessed by reviewing study protocols (if available) and comparing reported outcomes with the outcomes mentioned in the methods sections of the studies. Although a formal statistical test for publication bias (i.e., funnel plot) was not feasible due to the small number of included studies, we critically appraised the potential impact of bias on the cumulative evidence. The inclusion and exclusion criteria are provided in Table 1.

**Table 1:** Inclusion and Exclusion Criteria for Study Selection

Criteria Type	Criteria
Inclusion	Studies related to psilocybin in the context of long-haul COVID (e.g., post-COVID syndrome (PCS))
	Used keywords: “psilocybin,” “psychedelics,” “psychedelic-therapy,” “psychiatry,” “long-haul COVID,” “post-acute COVID-19,” “post-acute sequelae of SARS CoV-2 infection,” or “post-COVID syndrome”
	Published from January 2020 onward
	English-language publications
	Available in full text for review
	Any study design, as long as it included outcome data related to psilocybin use in long-haul COVID
Exclusion	Studies discussing psilocybin without a connection to COVID-19
	Duplicate publications
	Not written in English
	Studies not aligned with original search keywords or inclusion focus

## Review

The therapeutic potential of psilocybin for treating both psychological and physiological symptoms associated with long-COVID has garnered significant attention in recent research. Psilocybin, a serotonergic psychedelic, has shown promise in addressing the heightened mental health challenges brought on by the pandemic, as well as potential physiological impacts such as inflammation and cognitive impairment in patients with long-COVID. The following results synthesize findings from key studies that highlight the role of psilocybin in mitigating both the psychological and physical manifestations of long-COVID.

Psychological impacts and psilocybin’s mental health benefits

The COVID-19 pandemic triggered a substantial global mental health crisis, with many individuals experiencing heightened anxiety, depression, PTSD, and existential distress. This crisis has been particularly pronounced in vulnerable populations, such as frontline healthcare workers, individuals with preexisting mental health conditions, and those directly affected by COVID-19 or long-COVID [10,11]. The unique mental health challenges posed by long-COVID - including chronic anxiety, post-traumatic stress, and isolation - require novel treatment strategies. Psilocybin has shown promise in this regard, offering both rapid and long-lasting relief from mental health symptoms.

A pivotal study by Davis et al. [12] highlighted the benefits of psilocybin-assisted therapy in treating major depressive disorder (MDD). The randomized clinical trial demonstrated that psilocybin significantly reduced depression scores in individuals with MDD, with sustained benefits lasting for several months post-treatment. This is crucial in the context of the pandemic, where depression has become more prevalent, exacerbated by factors such as isolation, fear of illness, and uncertainty about the future. Psilocybin’s rapid antidepressant effects - achieved through a single or a few sessions - stand in contrast to traditional antidepressants, which can take weeks or months to exhibit efficacy. In a pandemic-induced mental health crisis, where the need for fast-acting treatments is critical, psilocybin provides a promising solution.

Similarly, Carhart-Harris et al. [13] conducted a comparative study involving psilocybin and escitalopram, a selective serotonin reuptake inhibitor (SSRI), for depression. Psilocybin not only proved more effective in reducing symptoms of depression but also showed greater improvements in emotional resilience, quality of life, and overall well-being. This study emphasizes psilocybin’s broader psychological benefits, which are particularly relevant for individuals experiencing long-COVID-related mental health challenges, including persistent feelings of hopelessness and despair. Psilocybin’s ability to provide holistic improvements beyond symptom reduction, such as fostering emotional resilience and a renewed sense of purpose, could be crucial for patients with long-COVID who struggle with prolonged psychological distress.

Dos Santos et al. [14] underscored the resurgence of psychedelics like psilocybin as potent tools in psychiatry, showcasing both immediate and prolonged therapeutic effects, especially when administered in supportive settings.

Cavanna et al. [15] explored the experiences of individuals using psychedelics during the early stages of the COVID-19 pandemic. Their findings revealed that many participants reported enhanced emotional resilience, a greater sense of connectedness, and an ability to process pandemic-related stress more effectively. This suggests that psilocybin not only helps alleviate psychological symptoms but also supports individuals in coping with ongoing challenges, such as isolation, uncertainty, and grief. These findings highlight the therapeutic potential of psilocybin for those grappling with long-term psychological impacts from the pandemic.

Moreover, Evens et al. [16] explored the experiences of individuals using psychedelics during the early stages of the COVID-19 pandemic. Their cross-sectional study revealed that many participants reported enhanced emotional resilience, a greater sense of connectedness, and an ability to process pandemic-related stress more effectively. This suggests that psilocybin not only helps alleviate psychological symptoms but also supports individuals in coping with ongoing challenges, such as isolation, uncertainty, and grief. These findings highlight the therapeutic potential of psilocybin for those grappling with long-term psychological impacts from the pandemic.

Psilocybin’s role in neuroplasticity and cognitive function

In addition to its mental health benefits, psilocybin’s ability to promote neuroplasticity makes it a strong candidate for treating cognitive impairments associated with long-COVID. Many patients with PCS experience “brain fog,” memory issues, and difficulties with focus and attention. These cognitive impairments have become one of the most persistent and debilitating symptoms of long-COVID, affecting patients’ ability to return to their normal lives.

Research by Shao et al. [9] has shown that psilocybin induces rapid and long-lasting growth of dendritic spines in the frontal cortex, which plays a critical role in cognitive flexibility and decision-making. Their study on mice revealed that a single dose of psilocybin increased spine density by 10%, and these changes persisted for over a month. This structural remodeling is believed to result in the cognitive and emotional benefits observed in psilocybin-treated patients. Increased synaptic density in the frontal cortex could counteract the synaptic loss associated with chronic stress and inflammation, which are prevalent in patients with long-COVID.

The cognitive benefits of psilocybin are further supported by Carhart-Harris et al. [17], whose six-month follow-up study found sustained symptom relief and cognitive improvement in patients with treatment-resistant depression, supporting the hypothesis that psilocybin may enhance cognitive function in long-COVID cases. Such neuroplastic and cognitive gains underscore psilocybin’s potential for aiding cognitive recovery in patients whose cognitive faculties have been compromised by long-COVID symptoms.

Psilocybin’s potential to address inflammation and immune dysfunction

COVID-19 is characterized by an overactive immune response, particularly in severe cases, often resulting in a “cytokine storm” that leads to widespread inflammation and tissue damage. This hyperinflammatory response can persist in patients with long-COVID, contributing to chronic fatigue, muscle pain, and cognitive deficits. Psilocybin’s anti-inflammatory properties make it a compelling candidate for addressing these immune dysfunctions. Khan et al. [18] conducted in silico studies showing that psilocybin derivatives have significant binding affinities to the SARS-CoV-2 main protease (Mpro) and interleukin-6 (IL-6) receptors, both of which are critical in the inflammatory process. The ability of psilocybin to inhibit IL-6 - a key driver of the cytokine storm in COVID-19 - suggests that it could help reduce inflammation in patients with long-COVID, potentially alleviating symptoms such as chronic pain and fatigue. This immune-modulating effect, combined with psilocybin’s psychological benefits, positions it as a multifaceted therapeutic agent for PCS.

Ona et al. [19] added that psychedelics like psilocybin may help reduce persistent psychological distress, which is often persistent in long-COVID cases. Their study revealed that regular psychedelic users exhibited lower levels of severe chronic psychological symptoms, further indicating that psilocybin may help address chronic inflammatory conditions seen in COVID-19 sequelae.

Psychedelic-assisted therapy in palliative care and long-COVID recovery

The application of psilocybin in hospice and palliative care settings has shown significant promise in alleviating psychological and existential distress, which is particularly relevant for patients with long-COVID. Many individuals suffering from long-COVID experience prolonged physical and emotional suffering, often with little hope for recovery. Psilocybin has been shown to alleviate these feelings of hopelessness and provide patients with a renewed sense of purpose and connection.

Ross et al. [4] demonstrated that a single dose of psilocybin significantly reduced anxiety, depression, and existential distress in patients with life-threatening cancer. This study underscores psilocybin’s potential to improve the quality of life for patients with long-COVID, many of whom experience chronic health issues and psychological distress. Similarly, a protocol by Ching et al. [20] on psilocybin therapy for obsessive-compulsive disorder (OCD) highlights psilocybin’s promise across mental health contexts. The reduction in existential anxiety and the promotion of spiritual well-being observed in these studies suggest that psilocybin could help patients with PCS cope with the chronicity and uncertainty of their symptoms.

Argento et al. [21] emphasized that psilocybin-assisted therapy could offer significant mental health benefits to long-COVID patients facing chronic distress and PTSD-like symptoms, supporting the potential of psilocybin as a therapeutic for those experiencing severe pandemic-induced trauma.

The role of self-medication with psychedelics during the pandemic

The COVID-19 pandemic also saw an increase in self-medication with psychedelics, including psilocybin, as individuals sought to manage their mental health in the absence of adequate healthcare resources. Matzopoulos et al. [22] examined the patterns of psilocybin use in the United States during the pandemic and found that many individuals turned to psychedelics as a coping mechanism for anxiety, depression, and existential distress. However, the lack of clinical supervision in these cases raises concerns about safety and efficacy, as unsupervised use of psychedelics can lead to adverse effects, particularly in vulnerable populations such as those with long-COVID. Ali et al. [23] also reported an increase in psychedelic use during the pandemic, as individuals sought alternative ways to manage their mental health. This reflects a broader societal shift towards the exploration of psychedelic substances as potential therapeutic agents. However, it also highlights the need for controlled clinical settings to ensure that these substances are used safely and effectively.

Future research directions: clinical trials and broader applications

While the current literature presents a promising case for psilocybin’s role in treating long-COVID, further research is needed to fully understand its therapeutic potential. Larger clinical trials are essential to determine the safety and efficacy of psilocybin in this context, particularly in comparison to existing treatments. Griffiths et al. [24] and Carhart-Harris et al. [17] have already demonstrated psilocybin’s safety in controlled clinical settings, but more studies are required to explore its specific application for patients with long-COVID. Pilot studies should focus on the use of psilocybin to treat specific long-COVID symptoms, such as chronic fatigue, brain fog, headaches, and muscle pain. These symptoms are some of the most persistent and debilitating for patients with long-COVID, and there is a need for novel approaches to address them. For example, small-scale clinical trials could compare the effectiveness of psilocybin-assisted therapy with conventional treatments for PCS, such as cognitive-behavioral therapy, SSRIs, or anti-inflammatory medications. Such studies could also explore the impact of psilocybin on both mental health and immune function, investigating how the drug’s neuroplastic and anti-inflammatory effects may contribute to symptom relief and long-term recovery.

Another critical area for future research is the exploration of psilocybin’s long-term effects on cognitive recovery in patients with long-COVID. Given the high prevalence of cognitive symptoms in this population, understanding how psilocybin can help rebuild cognitive function through enhanced neuroplasticity is paramount. Research by Shao et al. [9] suggests that psilocybin’s ability to promote dendritic spine growth and increase synaptic density could be particularly beneficial in this regard. Investigating these neuroplastic changes in patients with long-COVID, along with longitudinal studies to track cognitive improvements over time, will provide a clearer understanding of psilocybin’s role in cognitive rehabilitation. Kelly et al. [25] support these efforts, as their findings show a potential positive association between regular psychedelic use and cognitive resilience, which could inform recovery approaches for long-COVID.

In addition to clinical trials focused on long-COVID, broader research efforts should explore psilocybin’s potential to treat other pandemic-related mental health issues, such as PTSD and burnout in healthcare workers. Rucker et al. [26] found that 10 mg and 25 mg doses of psilocybin are well tolerated and did not have any adverse effects on cognitive function. Psilocybin’s efficacy in reducing symptoms of PTSD and promoting emotional resilience makes it a strong candidate for addressing the mental health needs of both COVID-19 survivors and those who have been on the frontlines of the pandemic response. Bouso et al. [27] underscore this necessity, advocating for psilocybin-assisted therapy as a possible remedy for chronic trauma and pandemic-related psychological distress.

Lastly, policy changes will be essential to expanding access to psilocybin-assisted therapy for patients with long-COVID and others suffering from pandemic-related mental health challenges. The reclassification of psilocybin from a Schedule I substance to one that can be used for medical treatment will open doors for more extensive clinical research and allow for greater integration of psychedelic therapies into mainstream healthcare. Marks and Cohen [28] discussed the need for regulatory reform to facilitate the wider acceptance of psilocybin and other psychedelics as legitimate therapeutic agents. Expanding access to these treatments, particularly for underserved populations affected by long-COVID, will require concerted efforts from both the scientific community and policymakers. Additionally, increased regulatory support will facilitate controlled studies, reducing risks associated with self-medication trends highlighted by Matzopoulos et al. [22], and ensuring safer, evidence-based therapeutic applications. A summary of the reviewed studies is provided in Table 2.

**Table 2:** Summary of Reviewed Clinical Studies Investigating Psilocybin-Assisted Therapy and Related Outcomes

Authors	Article Title	Study Group	Dose	Outcome
Grob et al. [10]	Pilot study of psilocybin treatment for anxiety in patients with advanced-stage cancer	12 patients with advanced-stage cancer and diagnosed with anxiety disorders (open-label pilot study)	Two experimental treatment sessions spaced several weeks apart. The subject was blinded to whether they were receiving niacin (250 mg) or active psilocybin (0.2 mg/kg)	Psilocybin administration was associated with reductions in anxiety and depression symptoms, improved mood, and enhanced spiritual well-being. These effects were sustained at a six-month follow-up
Davis et al. [12]	Effects of psilocybin-assisted therapy on major depressive disorder: a randomized clinical trial	24 adults diagnosed with major depressive disorder; Randomized, waiting list controlled clinical trial (Urn randomization)	Two psilocybin sessions: Session 1: 20 mg/70 kg; Session 2: 30 mg/70 kg with supportive psychotherapy administered two weeks apart.	Psilocybin-assisted therapy resulted in rapid and significant reductions in depressive symptoms, with 71% of participants showing a clinically significant response and 54% achieving remission at four weeks post-treatment.
Carhart-Harris et al. [13]	Trial of psilocybin versus escitalopram for depression	59 adults with moderate-to-severe major depressive disorder; Phase 2, double-blinded, randomized controlled trial	Psilocybin group: Two oral doses of 25 mg psilocybin, three weeks apart; Escitalopram group: daily 10–20 mg escitalopram for 6 weeks with two 1 mg “sham” psilocybin doses	The 16-item Quick Inventory of Depressive Symptomatology - Self Report (QIDS-SR-16) scores range from 0 to 27, with a higher score meaning greater depression; It was recorded at week 6 and compared to baseline. The study showed there was no significant difference in antidepressant effects between psilocybin and escitalopram with the psilocybin arm showing an 8-point reduction vs. a 6-point reduction in the escitalopram arm, although response/remission rates (71% vs. 48% response; 57% vs. 28% remission at week 6) favored psilocybin.
Shao et al. [9]	Psilocybin induces rapid and persistent growth of dendritic spines in frontal cortex in vivo	Adult C57BL/6J strain mice (40 males and 42 females)	1-2 mg/kg intraperitoneally	The study used chronic two-photon imaging to monitor dendritic spines in layer-5 pyramidal neurons in the medial frontal cortex of adult mice. Results showed a 10% increase in dendritic spine density and size in the medial frontal cortex of adult mice. Structural changes with increased spine formation were seen within 24 hours. This change was associated with amelioration of stress-related behavioral deficits and enhanced excitatory neurotransmission
Carhart-Harris et al. [17]	Psilocybin with psychological support for treatment-resistant depression: an open-label feasibility study	12 adults with moderate-to-severe, treatment-resistant depression (six men, six women); open-label feasibility trial	Two oral doses of 10 mg then 25 mg of psilocybin seven days apart, respectively, in a therapeutic setting	All patients tolerated dosing with only transient anxiety, confusion, nausea, or headache during sessions. Depressive symptoms were assessed from 1 week to 3 months after treatment with the 16-item Quick Inventory of Depressive Symptoms (QIDS). There were significant reductions in depression scores: by 1 week post-dose 2, the mean QIDS dropped by –11.8 points and at 3 months, the reduction was -9.2 points. There were marked and sustained improvements in anxiety and anhedonia
Ross et al. [4]	Rapid and sustained symptom reduction following psilocybin treatment for anxiety and depression in patients with life-threatening cancer: a randomized controlled trial	29 patients with cancer-related anxiety or depression were randomly assigned for treatment with psilocybin or niacin in a double-blind, placebo-controlled, cross-over trial	Single dosing session of 0.3 mg/kg psilocybin vs one single dosing session of 250 mg niacin (in conjunction with psychotherapy)	Along with psychotherapy, a single moderate dose of psilocybin produced rapid and long-lasting antidepressive and anxiolytic effects in patients with cancer-related distress. Along with decreased existential distress, increased spiritual wellbeing, improved quality of life and improved attitudes towards death. Effects lasting from 7 weeks to 8 months.
Griffiths et al. [24]	Psilocybin produces substantial and sustained decreases in depression and anxiety in patients with life-threatening cancer: a randomized double-blind trial	51 patients with life-threatening cancer experiencing clinically significant anxiety and/or depression; Randomized double-blind, cross-over trial	Low dose (1-3 mg/70kg) vs high dose (22-30 mg/70kg) of psilocybin administered in counterbalanced sequence with 5 weeks in between sessions and six-month follow-up. Session 1 occurred one month after baseline assessment. Session 2 occurred at five weeks. Returned for six-month follow-up	A single dose of psilocybin reduced anxiety and depression in this population for at least six months in 80% of the participants along with improvements in quality of life and decreased death anxiety
Rucker et al. [26]	The effects of psilocybin on cognitive and emotional functions in healthy participants: Results from a phase 1, randomised, placebo-controlled trial involving simultaneous psilocybin administration and preparation	20 healthy adult participants; Phase 1 randomized control trial	Single oral dose of 10-25 mg psilocybin or placebo administered simultaneously to up to six participants and in conjuntcions with one-to-one psychological support	Psilocybin significantly impaired cognitive flexibility and working memory but enhanced emotional empathy and positive mood compared to placebo

Discussion

The use of psilocybin in the United States has long been hindered by legal challenges, most notably its classification as a Schedule I substance under the Controlled Substances Act of the 1970s. This classification has significantly limited scientific research, as federal funding for studies on Schedule I substances is all but impossible to secure [28]. In the 1990s, some progress was made, such as research by the Maryland Psychiatric Research Center on LSD-assisted psychotherapy for substance use disorders [28]. However, without federal funding, researchers have had to rely on private foundations and industry sponsors, who often come with their own agendas. These private interests tend to focus on developing patentable drugs or therapies, placing control over research and treatment access in the hands of corporations. This creates potential conflicts of interest and restricts the availability of therapies. Additionally, the commercialization of psilocybin may exploit Indigenous communities who have historically used the substance, often without appropriate recognition or compensation.

The COVID-19 pandemic has further underscored the need for alternative mental health treatments. Lockdowns, social distancing, and quarantines led to widespread isolation, fear, and an increase in mental health disorders, along with higher risks of alcohol and drug abuse. Despite improvements in medical treatments and the development of vaccines, the pandemic continues to affect mental health globally. Given the limited availability of mental health professionals, alternative therapies such as psilocybin could play a crucial role in addressing these psychological challenges. Current literature suggests that larger-scale clinical trials are necessary to confirm psilocybin’s efficacy, but the early results are promising. Many studies indicate that, when administered under proper conditions, psilocybin is a safe and effective alternative therapy.

With the recent easing of restrictions on psilocybin research, studies have shown that the drug’s adverse effects are generally minor when supervised by healthcare professionals. Common side effects include tachycardia and mild dissociation, but these are typically well-managed in clinical settings. Future studies should incorporate similar safety protocols and aim to compare psilocybin-assisted therapy with other treatments, such as psychotherapy or SSRIs. Additionally, larger sample sizes and longer follow-up periods will be essential to fully understand the long-term effects of psilocybin.

A limitation of this review is its reliance on a limited number of databases, which may have introduced selection bias. The subjective nature of determining inclusion criteria for studies could also skew the results. Finally, the novel nature of psilocybin research means that only a small number of high-quality studies were available for review.

## Conclusions

Given the recent onset of the COVID-19 pandemic, research into psilocybin’s application for treating post-COVID syndrome (PCS) is still in its preliminary stages. This review, however, indicates that psilocybin shows potential in addressing COVID-19-related mental health conditions, including depression, anxiety, and mood disorders, and could also provide therapeutic benefits for healthcare providers and patients in palliative care contexts. Psilocybin’s inhibition of SARS-CoV-2 Mpro has shown promise in molecular simulations, though further in vitro and in vivo studies are required to substantiate these findings. Neuroplastic changes observed in animal models further support the compound’s potential therapeutic utility.

Despite these promising indications, regulatory barriers continue to restrict psilocybin’s availability for research and clinical application. The literature on psilocybin and COVID-19 remains nascent, and further rigorous studies are needed to establish its safety and efficacy. Future research should focus on psilocybin’s role in managing long-COVID, particularly given pharmacological evidence suggesting its potential to mitigate the cytokine storm associated with COVID-19. Although no clinical trials have yet explored psilocybin’s effectiveness in COVID-19 patients, double-blind trials comparing psilocybin to current standards of care, alongside pilot studies on symptoms like fatigue, mood dysregulation, headaches, and muscle pain, could provide a foundation for larger-scale investigations into its role in addressing the lasting effects of COVID-19.
